# Giant Hydatid Cyst Originating From Psoas Muscle Extending to the Iliac Bone: A Case Report

**DOI:** 10.1002/ccr3.70124

**Published:** 2025-01-21

**Authors:** Mohammad Hadi Niakan, Fatemeh Mirparsa, Hamid Zaferani Arani, Sedighe Hooshmandi, Elham Peyravi

**Affiliations:** ^1^ Trauma Research Center, Shahid Rajaee (Emtiaz) Trauma Hospital Shiraz University of Medical Sciences Shiraz Fars Province Iran; ^2^ Department of Health Policy, Faculty of Health, Scientific Pole of Health Sciences Education, Tehran Medical Sciences Tehran University of Medical Sciences Tehran Iran; ^3^ Department of Surgery Shiraz University of Medical Sciences Shiraz Iran; ^4^ Medical Imaging Research Center, Department of Radiology Shiraz University of Medical Sciences Shiraz Iran

**Keywords:** bone, *Echinococcus granulosus*, hydatid cyst, pelvis, psoas muscles

## Abstract

Hydatid cysts, caused by the *Echinococcus granulosus* parasite, predominantly affect the liver and lungs, but can also impact other organs such as the kidneys, brain, and muscles. Infection occurs when individuals ingest eggs from contaminated food or water, leading to cyst formation primarily in the liver. While hydatid cysts are commonly found in various endemic regions, muscular involvement is rare, particularly in the psoas muscle. This report presents a case of a muscular hydatid cyst, describes its management, and emphasizes the need for awareness and prompt intervention. A 50‐year‐old man presented with abdominal and left lower limb pain, along with weakness over several days. He was febrile, and a physical examination revealed pain during leg movement. Routine laboratory tests were normal. Ultrasound and computed tomography scans identified a solid cystic mass in the left lower quadrant, extending to the left lumbar muscle. After 28 days of preoperative albendazole therapy, the patient underwent surgery through a Rocky Davis incision, where a large hydatid cyst containing daughter cysts in the psoas muscle was discovered. The cyst was unroofed, and its contents were evacuated, followed by irrigation with hypertonic saline. Postoperative recovery was uneventful. He also received standard medication therapy with albendazole, and during monthly visits, no complications were observed during the one‐year follow‐up. This case contributes to the limited literature on muscular hydatid disease and highlights the importance of awareness among healthcare providers to ensure timely diagnosis and management.

**Summary**

- Hydatid cysts can affect rare sites, such as the psoas muscle, highlighting the need for a thorough investigation in unusual cases.
- A combination of clinical history, serological tests, and advanced imaging (CT/MRI) is crucial for accurate diagnosis.
- Complete surgical removal is the most effective treatment for symptomatic cysts, necessary to prevent complications like rupture and anaphylaxis.

## 1 Introduction

Hydatid cysts are parasitic infections that occur mainly in the liver and lungs [1]. Cysts can also affect other organs such as the kidneys, brain, and bones [2]. These cysts are caused by *Echinococcus granulosus*, a type of tapeworm, and dogs or other carnivores are considered definitive hosts, while sheep or other ruminants are intermediate hosts [3]. After eating food or water contaminated by dog feces containing parasite eggs, most of the embryos die in the liver capillaries, but some turn into cysts and even immigrate to other organs [1, 2]. Hydatid cysts are rarely (0.7%–0.9%) detected in muscle tissue, even in endemic countries [3]. The psoas muscle is a rare site for hydatid cysts, and there is little knowledge about its management. Although a lack of proper management could lead to mortality, open surgery is preferred and can be a curative approach for multiple and large liver hydatid cysts [4]. Also, regular follow‐up is required to detect recurrence [4]. To the best of our knowledge, only 16 cases of muscular hydatid cyst infection [5, 6, 7, 8, 9, 10, 11, 12, 13, 14, 15, 16, 17] were reported in Iran. Hence, in this report, we present a patient with muscular hydatid disease and its management.

## 2 Case History/Examination

A 50‐year‐old man presented with complaints of abdominal (left lower and left upper quadrant) and left lower limb pain, as well as weakness, from 27 days ago that worsened gradually until the day of the attendant. There were no past medical diseases, past surgical histories, or drug histories. Also, he and his family had a history of caring for animals such as sheep, goats, and dogs, as well as a residency in Bushehr city (Bushehr province), located in southwestern Iran, which is endemic for hydatid cysts. In the physical examination, he had a fever (temperature: 38.5) and pain in active and passive flexion of the left lower extremity and abdominal left lower quadrant tenderness.

## 3 Methods

**FIGURE 1:**
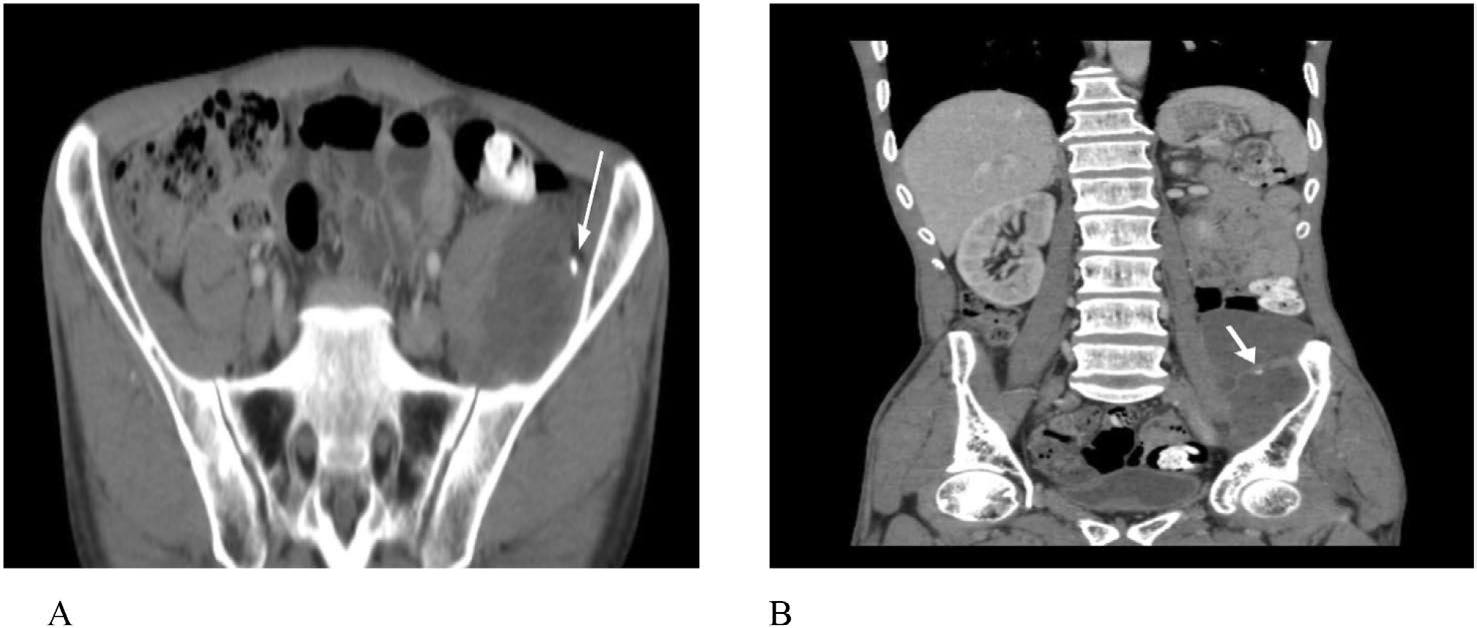
Spiral CT scan of abdomen and pelvis with IV and oral contrast administration. (A) Axial contrast‐enhanced CT image shows thin wall cystic mass in Lt lower quadrant of the abdominal cavity with involvement of Lt iliacus muscle, and foci of calcification in the wall are seen (arrow). (B) Coronal contrast‐enhanced ct image shows a multilocular thin wall cystic mass in the LLQ containing multiple thin septa and foci of calcification in the septa (arrow) with peripheral thin‐rim enhancement in the wall and septa, without a solid component; no free fluid in the abdominopelvic cavity is seen.

According to our patient's signs and symptoms, there were some differential diagnosis such as hydatid cyst, abdominal abscess, cystic teratoma, cysticercosis (pork tapeworm infection), peritonitis, abdominal sepsis, and tuberculosis (TB). The routine laboratory tests in our patient were normal. Ultrasound showed evidence of a hyperechoic solid cystic structure measuring 86 × 36 mm in the left lower quadrant of the abdomen, indicating the formation of a collection. The computed tomography (CT) scan showed a cystic mass measuring 71 × 43 × 128 mm on the left side of the abdominal pelvic cavity, which extended to the left lumbar muscle. Cystic lesion in the liver and lung was not seen (Figure 1).

**FIGURE 2:**
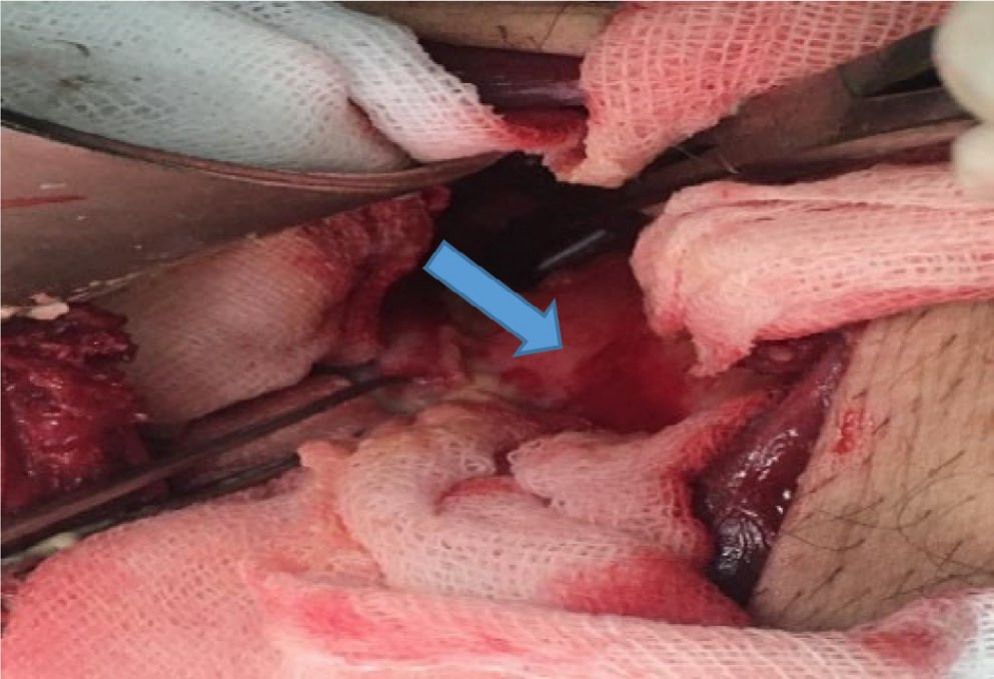
Intraoperative picture of a hydatid cyst (thick blue arrow) that extends from the psoas muscle to the iliac bone.

**FIGURE 3:**
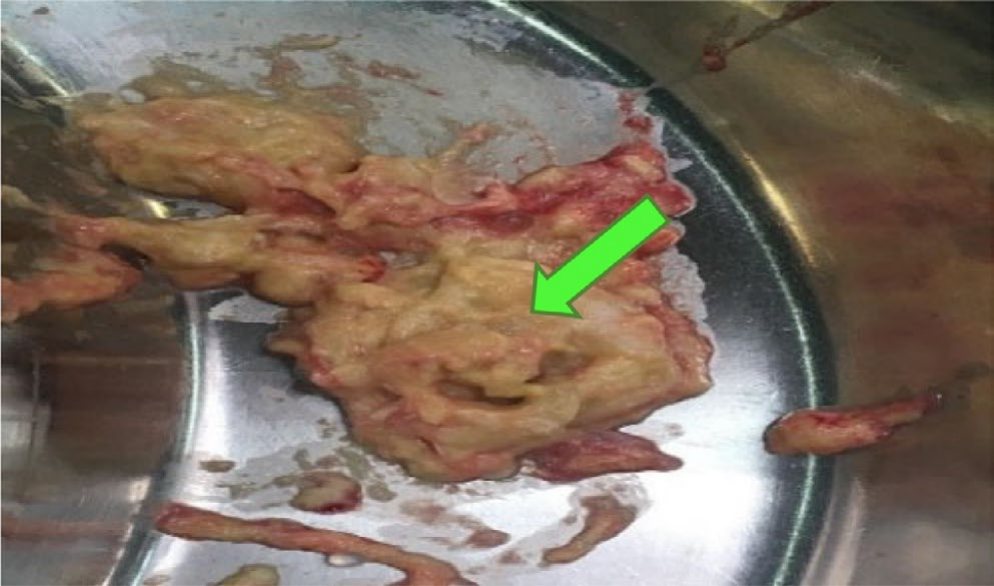
Hydatid cyst removed (thick green arrow). A giant hydatid cyst containing daughter cysts measuring about 40 × 50 × 110 mm was found in the psoas muscle on the iliac bone.

Regarding our first diagnosis, hydatidosis, the patient received albendazole (400 mg, every 12 h/for 28 days), and then underwent surgical cyst removal [18]. Briefly, via general anesthesia, the patient was placed in the supine position, and a Rocky Davis incision in the left lower quadrant of the abdomen was performed. We entered the retroperitoneal space and dissected the left psoas major muscle (in level of L5 vertebrae). A large hydatid cyst containing daughter cysts measuring about 40 × 50 × 110 mm was found in the psoas muscle on the iliac bone (Figure 2). The cyst was unroofed, and all content was evacuated (Figure 3); the laminated and germinal layers were partially removed and sent for pathology. Then, the cyst was irrigated with hypertonic saline. After hemostasis, a mushroom drain was inserted into the site of the drained cyst and fixed to the skin with silk 2.0. Then, the abdominal muscles were approximated with vicryl 2.0, and the external oblique fascia was closed with vicryl 0. The skin was closed in a vertical mattress manner with Nylon 3.0, and a sterile dressing was applied. Histopathological examination confirmed hydatidosis.

The patient was discharged after 3 days with good general conditions and received a standard medication regimen (albendazole tablet 400 mg orally twice a day for two 28‐day cycles, separated by 14 drug‐free days).

## 4 Discussion

Hydatid cyst disease is one of the important diseases of humans and animals, and, according to the World Health Organization, it is endemic in many parts of the world, such as the Mediterranean Region, the Middle East, South America, Central Asia, and Iran [5].

The hydatid cysts, caused by the *Echinococcus* parasite, are particularly prevalent in Iran, with higher incidence rates observed among women aged 30–49, especially in rural communities [5]. Although this is not consistent with the demographic characteristics of our patient, there is a report of the disease in middle‐aged men [18].

Hydatidosis is more prevalent among nomadic communities in Bushehr province, Iran, due to their proximity to dogs, the definitive host of *E. granulosus* [19]. Bushehr province is known for its animal husbandry and agriculture, with a significant population of tribal nomads residing in the area [11]. The case discussed in this study involved a rural resident living in Bushehr province, where the hydatid cyst is endemic. Also, he had a history of contact with dogs, particularly as a pet, as well as sheep and other livestock.

Human infection occurs when individuals inadvertently ingest eggs shed in the feces of infected dogs, as these eggs can survive in the environment for weeks [19, 20]. Once ingested through contaminated food or water, the eggs hatch in the small intestine, allowing the parasites to enter the bloodstream and spread to various organs, including the liver and lungs, where they develop into larval cysts [13]. This delayed transmission underlines the need for awareness and preventive measures in affected areas.

Hydatid disease primarily affects the liver, accounting for 75% of cases, while the lungs are involved in 15% of cases [21, 22]. Other organs, including those in the abdominal cavity, pelvis, and nervous system, are affected in only 10% of instances [22]. Clinical presentations can vary based on the location of the cysts. For example, a cyst located in the pelvic cavity may cause abdominal swelling, pain, nausea, vomiting, and low blood pressure [8]. In severe cases, a ruptured cyst can trigger an anaphylactic reaction, presenting with hypotension, syncope, and fever [9]. A critical complication of cyst rupture is the potential implantation of daughter cysts in other body areas, which can lead to organ failure, increased morbidity, and mortality [18]. Awareness of these clinical manifestations is essential for timely diagnosis and management of hydatid disease.

Regarding Table 1, some of the most recent important cases of muscular hydatid cysts are presented [17, 18, 23, 24, 25]. As mentioned in the literature, diagnosis of hydatid disease involves a comprehensive approach that includes patient history, physical examination, laboratory tests, and imaging studies [26]. The most effective imaging modalities for identifying cystic echinococcosis are CT scans and MRIs, as they provide detailed visualizations of cystic formations in affected organs [25]. Laboratory tests, including serological assessments for specific antigens related to cystic echinococcosis, are also crucial [26]. These tests exhibit a sensitivity of 80%–100% and specificity of 88%–96% for liver involvement, but their efficacy diminishes in cases affecting the lungs (50%–60% sensitivity) and other organs (25%–56% sensitivity) [27]. Because imaging techniques are generally more sensitive than serological tests, a negative serology should not preclude further investigation using routine scans.

**TABLE 1:** Previous reports of muscular hydatid cysts.

Authors	Patient and clinical findings	Diagnosis	Management	Outcomes
Zendeoui et al. 2024 [18]	A 40‐year‐old man with persistent lumbosciatic pain	Cyst in the left thigh	Surgical excision through the intermuscular septum between the biceps femoris and semitendinosus muscles	Resolution of symptoms
Arab et al. 2024 [23]	A 40‐year‐old woman with fatigue and abdominal pain	Two cysts in the left psoas major muscle	Cystectomy via the left pararectal approach with retro‐peritoneal passage	Clinical improvement
Jia et al. 2023 [24]	A 62‐year‐old woman with intermittent pain in her right lower limb and numbness in the sole of her right foot	Cyst in the posterior tibialis and soleus muscle spaces behind the right calf	Open surgery and post‐operation medicine therapy	Complete removal of the cyst
Agholi et al. 2023 [17]	A 40‐year‐old woman with a soft, mobile, and non‐tender lump in her left upper arm	Cyst in triceps brachii	Open surgery and post‐operation medicine therapy	Symptom improvement without recurrence
Abhishek et al. 2012 [25]	A 60‐year‐old woman with swelling in the right paraumbilical region	A cystic lesion adherent to the peritoneum without any intraperitoneal extension	Decompressive surgery and post‐operation medicine therapy	Significant symptom relief

Consistent with previous reports [5, 6, 7, 8, 9, 10, 11, 12, 13, 14], in our patient according to clinical findings and initial ultrasound results, the patient underwent additional imaging examinations, that is, CT scan, and a muscular hydatid cyst was considered as the first and most probable diagnosis.

For symptomatic cysts, complete surgical removal remains the most effective treatment option [17, 18]. Also, currently, medical treatment with albendazole after surgery is a standard of care and consistent with previous reports [23, 24, 25]; our patient received it and during 1 year of postoperative follow‐up, no recurrence or complications were observed.

## 5 Conclusion and Results

In the reported patient, the result of the treatment was satisfactory, and the symptoms completely improved after treatment. During monthly follow‐up visits (for 12 months), there was no complications and/or evidence of recurrence.

## Author Contributions

**Mohammad Hadi Niakan:** conceptualization, data curation, investigation, methodology, writing – review and editing. **Fatemeh Mirparsa:** investigation, software, visualization, writing – review and editing. **Hamid Zaferani Arani:** methodology, validation, visualization, writing – original draft. **Elham Peyravi:** methodology, supervision, validation, visualization, writing – original draft, writing – review and editing.

## Ethics Statement

The research obtained approval from the School of Medicine of Shiraz University of Medical Sciences (Number: 2024/085). The participant in the study was a volunteer who had given informed consent to the study.

## Consent

The patient had given informed written consent to report the individual patient data.

## Conflicts of Interest

The authors declare no conflicts of interest.

## Data Availability

The clinical documentation of the presented case cannot be made public due to the detailed, identifiable information of the patient.
